# Recognition of Higher Order Patterns in Proteins: Immunologic Kernels

**DOI:** 10.1371/journal.pone.0070115

**Published:** 2013-07-29

**Authors:** Robert D. Bremel, E. Jane Homan

**Affiliations:** ioGenetics LLC, Madison, Wisconsin, United States of America; University of Ulm, Germany

## Abstract

By applying analysis of the principal components of amino acid physical properties we predicted cathepsin cleavage sites, MHC binding affinity, and probability of B-cell epitope binding of peptides in tetanus toxin and in ten diverse additional proteins. Cross-correlation of these metrics, for peptides of all possible amino acid index positions, each evaluated in the context of a ±25 amino acid flanking region, indicated that there is a strongly repetitive pattern of short peptides of approximately thirty amino acids each bounded by cathepsin cleavage sites and each comprising B-cell linear epitopes, MHC–I and MHC-II binding peptides. Such “immunologic kernel” peptides comprise all signals necessary for adaptive immunologic cognition, response and recall. The patterns described indicate a higher order spatial integration that forms a symbolic logic coordinating the adaptive immune system.

## Introduction

The adaptive immune system is capable of cognition, coordinated activation, and memory recall. It differentiates self from non-self and reacts to novel or exogenous epitopes through the integrated action of antibody and cell-mediated responses. The interplay of multiple coordinated signals controls the level of reaction. Pattern recognition capabilities comprise both stochastic components (B-cell receptors, T-cell receptors, and antibody binding) and genetically controlled components (MHC binding). Diverse aspects of the coordination needed to mount and recall an adaptive immune response have been described extensively in the literature over decades, among them the role of T-cell help (T_H_) to B-cells [1], epitope-directed processing by B-cells [2], the ability of dendritic cells to store epitope peptides and re-present them to B-cells [3], [4], cross presentation by dendritic cells [5], [6], and the necessity of T_H_ cells in establishing CD8+memory [7] and to provide help for B-cell memory recall [8]. Serine protease with trypsin-like specificity facilitates uptake of epitope peptides by B-cells [9], [10]. Cleavage by asparagine endopeptidase is critical for opening up protein structures to enable subsequent enzymatic activity to release MHC binding peptides [11]. The cathepsin peptidases have diverse roles in immune processing [12]. Physical proximity of B-cell linear epitopes and cognate T-cell help has been engineered into small synthetic peptides [13], [14] and observed in various viral proteins [15]–[18]. Meta-analysis has noted frequent reporting of a peptide as a T-cell epitope by one laboratory but as a B-cell epitope by another [19]. Reports of coincidence of all three elements: B-cell epitope, MHC-I and MHC-II, are rare [20]. A systematic characterization of the spatial relationship of the epitope components within a protein has, however, been lacking.

We recently described the application of the principal components of amino acid physical properties (PCAA) to predict the binding affinity of peptides to MHC-I and MHC-II molecules of numerous alleles and the probability of peptides binding B-cell receptors [21], [22]. In examining graphic plots of the location of predicted high affinity MHC binding proteins and B-cell epitopes in many proteins, we noted the frequent occurrence of “coincident epitope groups” in which multiple classes of epitope appear to overlap [21]–[23]. Recently, new proteomic approaches have provided a means to deduce large numbers of enzymatic cleavage patterns in a single experiment [24], [25]. Included in the datasets thus generated are the cleavage patterns of several peptidases, including human cathepsin B, L, and S, shown to be important in antigen processing by genetic knockout and enzyme inhibitor studies [26]. We applied PCAA prediction methods using these datasets to derive discriminant equations for the prediction of probability of cleavage of primary amino acid sequences of proteins by human cathepsins B, L and S (Bremel and Homan, unpublished information; see File S1). This now enables us to combine these predictive methods to determine the spatial relationships between cleavage by these cathepsins, high probability B-cell epitope contact points, and predicted high affinity MHC-I and MHC-II binding peptides for multiple alleles.

## Results

Throughout this paper we use the term “proximal” to denote a position relatively nearer to the N-terminus of a protein and “distal” for positions nearer to the C-terminus.

We applied discriminant equation ensembles developed using PCAA to predict the probability of human cathepsin L and S cleavage sites in tetanus toxin (gi: 40770, 1315 amino acids), a protein which has a high frequency of experimentally documented T-cell and B-cell epitopes [27]–[29] (see Figure S1). The output was compared with predicted MHC-I and MHC-II binding affinity and probability of B-cell binding. Resultant data sets are provided in Table S1. We applied the same analysis to ten additional bacterial, viral, mammalian, and plant proteins. Further correlations were then conducted to examine positional relationships between B-cell epitopes and MHC-I and MHC-II binding peptides.

Several statistical procedures commonly used to analyze equally-spaced data points in time series were applied to analyze patterns in several metrics derived from the primary amino acid sequences of proteins shown in Table 1. A primary tool for delineating periodicities in a data series is the spectral density, in which a statistical test is made of the probability of a pattern having arisen randomly or an underlying periodicity in the data series.

**Table 1:** Fisher’s Kappa statistic test p-values for presence of periodic components in protein sequences.

Protein and gi	Asn	hCAT_L	hCAT_S	BEPI Score	A*02∶01 #	DPA1*02∶01-DPB1*01∶01 #	DRB1*01∶01 #	z1	z2	z3
Mumps hemagglutinin neuraminidase JerylLynn Minor 19070176	0.6362	0.0436	0.0297	<0.0001	0.0795	<0.0001	<0.0001	0.0781	0.4559	0.7589
*Staph. aureus* Cell surfacereceptor IsdB 19528514	0.6852	0.6063	0.7082	<0.0001	<0.0001	<0.0001	<0.0001	0.4004	0.0143	0.4547
*Staph. aureus* Cell surfacereceptor IsdH 19528514	0.2654	0.5401	0.2531	<0.0001	<0.0001	<0.0001	<0.0001	0.2569	0.0217	0.2335
Foot-and-mouth diseasevirus P1 polyprotein311701499	0.5117	0.9310	0.3936	<0.0001	0.0843	<0.0001	<0.0001	0.6068	0.8342	0.6877
Diphtheria toxin 38232848	0.5959	0.3927	0.1078	<0.0001	0.0055	<0.0001	<0.0001	0.3168	0.7183	0.3632
Tetanus toxin precursor40770	0.1316	0.2822	0.2270	<0.0001	0.0115	<0.0001	<0.0001	0.2736	0.9340	0.4037
Human coagulation factorVIII isoform a 4503647	0.8849	0.1489	0.0519	<0.0001	<0.0001	<0.0001	<0.0001	0.0021	0.7745	0.6098
*Brucella melitensis*polynucleotide phosphorylasepolyadenylase 17988244	0.9047	0.0166	0.2560	<0.0001	0.0388	<0.0001	<0.0001	0.1226	0.8827	0.4628
*Brucella melitensis*methionine sulfoxidereductase B 17989164	0.9602	0.5138	0.7207	0.0033	0.3423	0.0003	<0.0001	0.9082	0.2105	0.8364
*Arachis hypogaea* Arah 6 allergen 57118278	0.3927	0.0574	0.0498	<0.0001	0.3968	<0.0001	<0.0001	0.0154	0.3264	0.5591
*Arachis hypogaea* LTPisoallergen 1161087230	0.1465	0.7434	0.6271	<0.0001	0.0127	<0.0001	<0.0001	0.6978	0.3041	0.4159

#:representative alleles are shown, all were analyzed.

Fisher’s Kappa statistic that tests the null hypothesis that the values in the series are drawn from a normal distribution with variance 1 against the alternative hypothesis that the series has some periodic component. Metrics tested: Asparagine endopeptidase, human cathepsin L and human cathepsin S cut sites, B-cell epitope contact probability, predicted MHC-I and MHC-II binding affinity principal components of amino acids z1, z2, z3.

Statistical tests for the predicted cathepsin L and S cleavage site probabilities, and asparagines, as a target for asparagine endopeptidase (AEP), showed no statistically significant periodicity and thus are randomly distributed within the primary sequence of all 11 proteins. Likewise, the physical properties of amino acids, as indicated by the principal component vectors (z1, z2, z3), are mostly randomly distributed. However, there are some statistically significant patterns predicted with modest levels of significance (p<0.01–0.002), indicating they show at best weak periodicity or could be artefactual. In contrast, MHC-II alleles, as represented in Table 1 by DRB1*01∶01 and DPA1*02∶01/DPB1*01∶01, showed strong periodicities in each of the proteins, as do predicted B-cell linear epitope contact points (i.e. antibody contacts). For these two variable classes the probabilities for rejection of the null hypothesis ranged from 10^−9^–10^−50^. Individual MHC-I alleles, as represented in Table 1 by A*02∶01, showed statistically significant periodicities only in some proteins, a characteristic common to all MHC-I alleles analyzed (not shown). Examples of the periodograms for tetanus toxoid are found in Figure S2.

The strong periodicities observed led us to explore the cross-correlations among the immunological features in the primary amino acid sequences. A cross-correlation coefficient was computed between the data elements of two series of metrics, across a series of amino acid positions with their positive and negative flanking regions (lags) of ±25 amino acids. We performed pairwise cross-correlation analysis using the cathepsin L and cathepsin S cleavage probability predictions, the standardized MHC peptide binding affinity predictions for 74 MHC-I and MHC-II alleles from humans and mice, and the predictions of B-cell binding points. This effectively superimposes all pairs of metrics from every amino acid position in the complete protein into one vector of numbers. The strength and spatial separation of the relationships between the metrics are shown by the magnitude of the correlation coefficients of the various lag positions. The resulting correlation signals at the various lags were striking, indicating that not only are the individual patterns repetitive, they also have specific interrelationships. We present the results for tetanus toxin here; results for the additional proteins were entirely consistent with the findings for tetanus toxin and are provided in Figures S3.1–S3.5.

### Cathepsin Cleavage Frequencies

**Figure 1:**
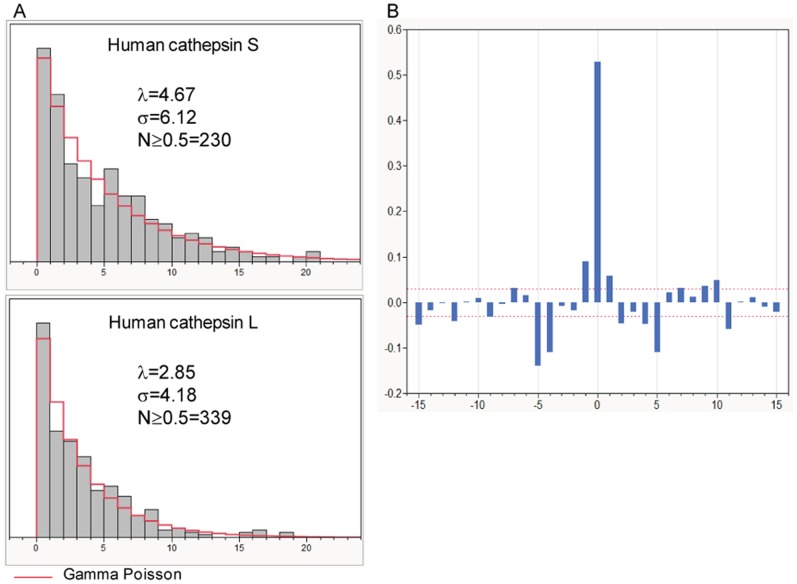
Predicted cleavage of tetanus toxin by human cathepsin L and S. A: Shows the distribution of the distance between successive cleavage probabilities of ≥0.5 for the two cathepsins. λ = expected value (mean) and σ = over dispersion (variance) of the fitted gamma Poisson distribution. B: Cross correlation of cleavage by cathepsin L and cathepsin S cleavage probabilities. A high correlation centered at zero indicates that the two cathepsins have a tendency to cut at the same site within the protein. This is flanked by high correlation of low probability of cleavage at ±5 amino acids of the initial cleavage. The red dashed lines at ±0.04 indicate the 95^th^ percentile confidence limits for non-significant correlation; values outside this band are significant p<0.05.

Cathepsin L and S are endopeptidases found in the endosome of antigen presenting cells. Differential levels of expression have been shown in B-cells, dendritic cells, macrophages and thymic epithelial cells [30], [31]. Of the several peptidases known to be located in endosomes, gene knockout and enzyme inhibitor studies of cathepsin L and S have shown that these two peptidases are critically involved in antigen processing [26], [30], [32]–[34]. However, cathespsin B, an exopeptidase, appears not to be essential. Cathepsin L and S are predicted to cleave target proteins frequently and exhibit a γ Poisson distribution of distance between adjacent cleavage points. We predict that cathepsin L will cleave (predicted probability of cleavage ≥0.5) tetanus toxin 339 times with a mean distance (λ) of 2.85 amino acids between scissile bonds. Cathepsin S is predicted to cleave less frequently (230 times, λ = 4.67). The distribution of high probability cleavage sites is shown in Figure 1A. Our underlying predictions are built on vectors encoding the cathepsin preferences for cleavage site octomers [35]. Beyond the requirement for the octomers, the overall within-protein patterns of cathepsin L and cathepsin S cleavage in the proteins tested were shown to be random (see Table 1 and also Figure S2 panels K and L). Figure 1B shows that the predicted cleavage points for cathepsin L and cathepsin S are highly correlated. Figure S3.1 shows this correlation for all eleven proteins studied. The strong association of cleavage by cathepsin L and S at the same scissile bond is coupled with weaker positive correlations at ±1 from that position that is consistent with the nested peptides often seen in experimental work [36], [37]. There is a statistically significant correlation of low probability of cleavage at amino acid positions ±4 and ±5. Taken together, the implication is that the next cleavage occurs where an appropriate cleavage site octomer combinatorial sequence is present, but that it will occur somewhere more than ±5 amino acid positions from the first cleavage. Given the close correlation of cathepsin S and cathepsin L, for brevity further descriptions below will focus on cathepsin L. All of the patterns we describe for cathepsin L are generally the same as seen with cathepsin S, although they differ in detail. Further, the training sets used for developing the discriminant equations consisted of peptides generated by both trypsin and Glu-C and thus give higher confidence predictions particularly for peptides containing lysine and arginine [25]. Consistent with its different cleavage preferences, cathepsin B does not exhibit the same type of patterns (Figure S3.1).

### Correlation of Predicted MHC Binding Affinity to Cathepsin Cleavage

**Figure 2:**
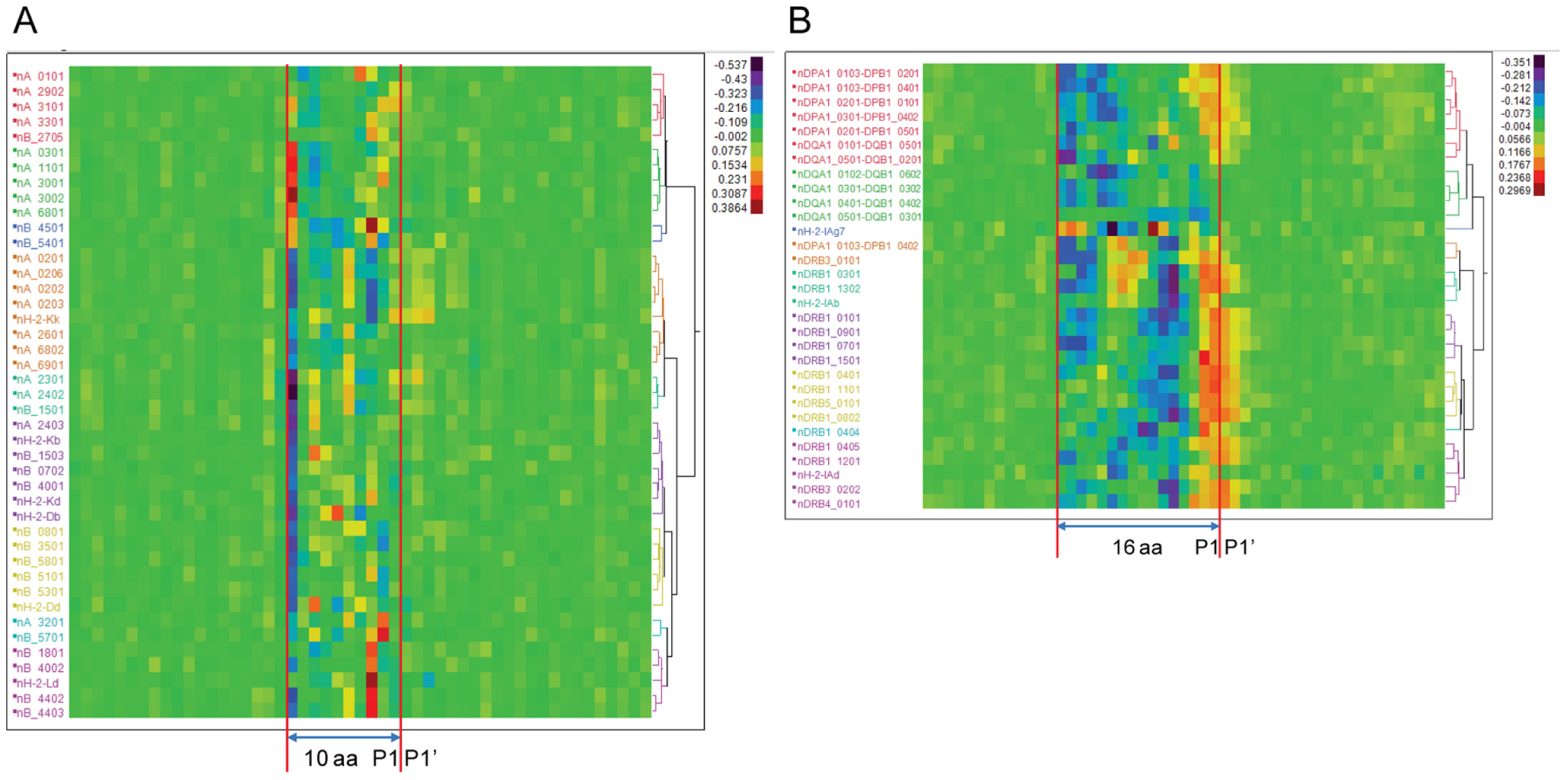
Heat diagrams of cross correlation of predicted MHC binding with predicted cathepsin L cleavage in tetanus toxin. The predicted binding affinity of sequential 9-mers (A: MHC-I) and 15-mers (B: MHC-II) for different human and murine MHC alleles is shown correlated with predicted cathepsin L cleavage sites. As the natural log of MHC binding affinity has been standardized to a zero mean and unit variance by allele within protein, the highest affinity has the lowest numerical value (blue on the thermometer scale). Human cathepsin L cleavage probability ranges from 0–1. The magnitude and sign of the correlation coefficient for each allele is indicated by the thermometer scale. The 95^th^ percentile confidence limits for non-significant correlations is ±0.05. By convention, cleavage is designated as occurring at the P1-P1’ scissile bond; this position is marked. For cathepsin L and S the amino acid at position P2 has a strong tendency to be more hydrophobic than P1. Predicted MHC-I high affinity binding peptides align with their index positions at 10 amino acid positions proximal (toward N-terminus) of the P1P1’and MHC-II at 16 amino acids proximal of P1P1’. The corresponding plot for all 11 proteins is shown in Figure S3.1.

**Figure 3:**
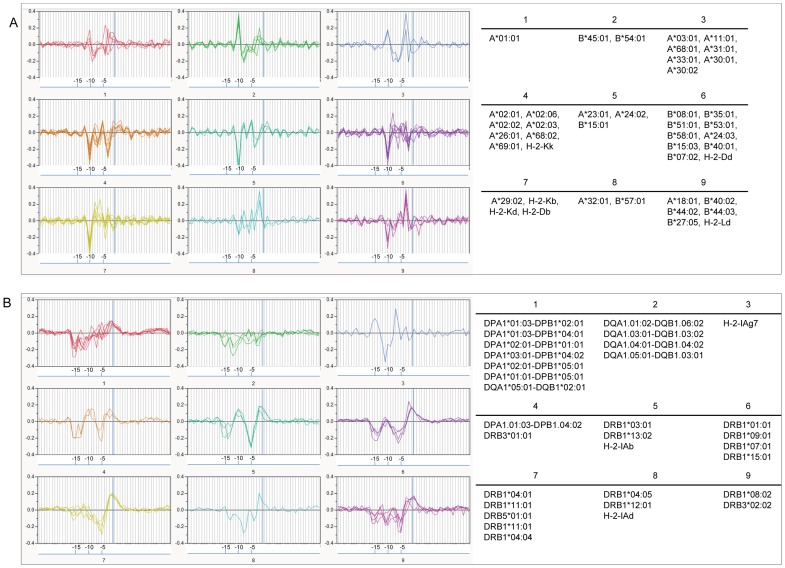
Parallel plots of cross correlation of predicted MHC binding with cathepsin L cleavage for clusters of alleles in tetanus toxin. The cross-correlation hierarchies of Figure 2 are shown separated by allele clusters to differentiate their patterns. The blue vertical line marks the P1P1’ cathepsin scissile bond position. The numbering of the X axis reflects amino acid positions proximal of the human cathepsin L cleavage site. The 95 percentile confidence limits differ for each panel, but range from ±0.02–0.05 and are not shown for clarity. Thus the prominent peaks in the graphs are statistically significant but the smaller oscillations of the graphics around zero are not. The corresponding plot for all 11 proteins is shown in Figure S3.2.

Given the documented relationship of cathepsin L and cathepsin S to MHC peptide loading, we then cross-correlated predicted cathepsin L scissile bond probabilities with the predicted MHC-I and MHC-II binding affinity of all 9-mer and 15-mer peptides, indexed by a single amino acid displacement across the entire protein sequence. The binding affinity data was standardized to zero mean and unit variance within protein to eliminate scale effects. Figure 2 shows the hierarchical clustering based on predicted binding affinity by allele (65 HLA and 9 murine), first of MHC-I (Figure 2A) and secondly of MHC-II (Figure 2B) relative to the predicted cleavage site. A striking relationship between the high affinity MHC binding peptides and cathepsin L cleavage is clearly seen in the heat diagrams (Figure 2). A majority of MHC-I allele high affinity binding peptides align with their index position located 10 amino acids proximal of the predicted cathepsin L scissile bond. When each allelic cluster is examined individually (Figure 3A), we see a characteristic pattern of highest binding affinity with a lag proximal of the cleavage site predominantly at 10 amino acids, but at position 8 and 6 amino acids proximally for some alleles. We also examined alignment as a result of processing using the 20S proteasome provided by Netchop [38] and found the output essentially consistent (shown in Figure S4). For MHC-II (Figures 2B and 3B) alignment occurs predominantly at position 15 or 16 proximal of the cleavage site, with a secondary peak of alignment at position 5 or 6. As MHC-II binding peptides are longer they span multiple potential cathepsin L cleavage sites. Hence, taking into consideration an “exclusion zone” of low cathepsin L cleavage probability ±5 amino acids either side of a cleavage as described above, the secondary peak reflects the next distally available cathepsin L cleavage site, i.e. 10 amino acids beyond the initial aligned scissile bond. The distribution patterns do not indicate any correlation of MHC binding distal to cathepsin cleavage sites, indicating that the role of cathepsin L is predominantly at the C-terminus of MHC binding peptides.

### B-cell Epitopes and Cathepsin Cleavage

**Figure 4:**
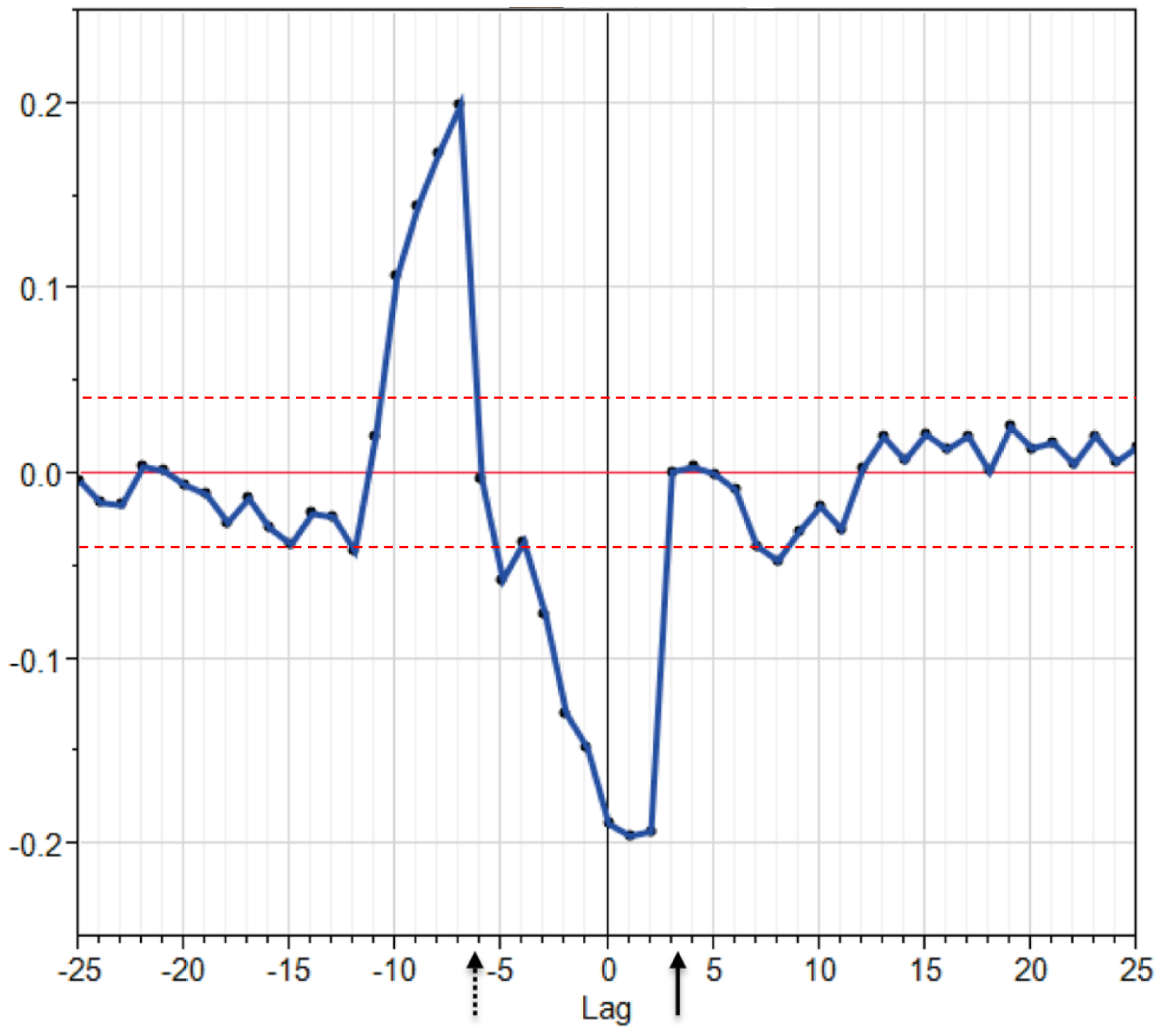
Cross correlation of cathepsin L cleavage probability and B cell epitope probability in tetanus toxin. Index position zero corresponds to the N-terminal amino acid (P4) of the cleavage site octomer of cathepsin. Hence the scissile bond P1-P1’ occurs at positions 3–4 (solid arrow). The B-cell epitope prediction algorithm evaluates each amino acid in the context of the 4 amino acids each side hence showing the probability that the center amino acid of a 9-mer is a B epitope contact point that will be at index position zero in this graphic. The predictions suggest a strongly negative correlation with cathepsin cleavage to amino acid position running from the predicted cleavage point to -6 (dashed arrow), or that the probability of the peptide whose N terminus is at the position is not favorable for cutting by the peptidase in this region. The dashed lines at ±0.04 indicate the 95^th^ percentile confidence limits for non significant correlation; values outside this band are significant p<0.05.The corresponding plot for all 11 proteins is shown in Figure S3.3.

We next cross-correlated B-cell linear epitope binding probability with cathepsin L cleavage probability. The B-cell epitope prediction algorithm evaluates each amino acid in the context of the 4 amino acids each side hence showing the probability that the center amino acid of a 9-mer is a B epitope contact point [21], [39], [40]. In this computation, the B-cell contact point is set at zero and the scissile bond (P1-P1’) is between+3 and+4. Figure 4 shows a strong negative correlation immediately proximal of the scissile bond (position+3 to −6) and a positive correlation proximal of the B-cell epitope contacts at positions -7 to -11. The differences in the correlation coefficients are statistically significant (±0.2 compared to the 95% confidence limit of non-correlation of approximately ±0.04). Hence there appears to be a high probability of cathepsin L cleavage immediately proximal to a B-cell epitope, but an exclusion zone of approximately 9 amino acids across a B-cell epitope which is protected from cathepsin L cleavage.

### Correlation of B Cell Binding to MHC Binding

**Figure 5:**
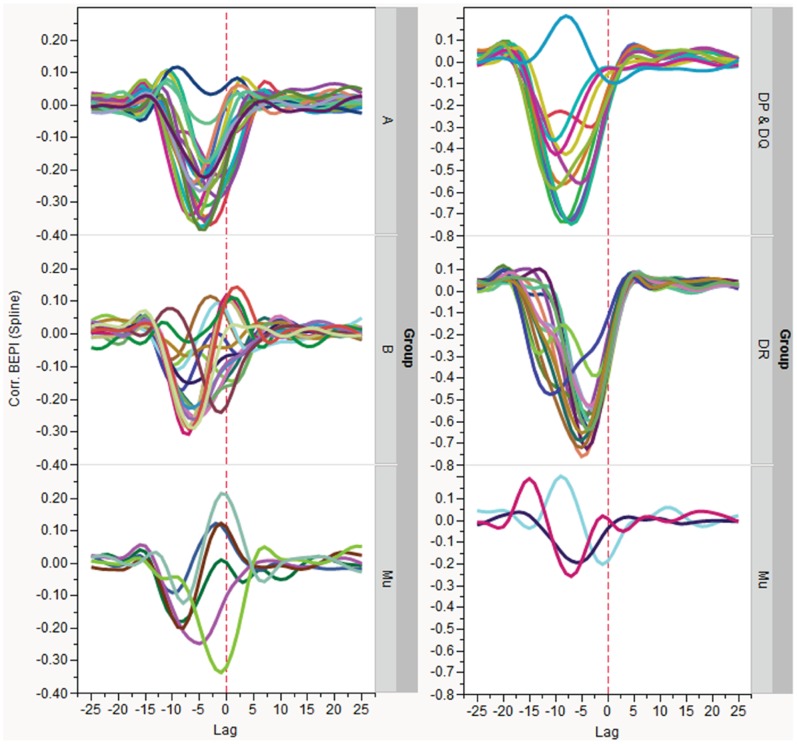
Inverse cross correlation of B cell epitope contact positions with N terminal position of predicted MHC binding peptides in tetanus toxin. Panel A shows (top to bottom) correlation of MHC-I, Class A, Class B, and Murine. Panel B shows correlation of MHC-II, top to bottom DP and DQ, DR, and murine alleles. Each allele is represented by a colored line. The natural log of MHC binding affinity has been standardized to a zero mean and unit variance by allele within the protein and thus the highest affinity has the lowest numerical value. Highest correlation (negative sign is consistent with increased affinity) varies in lag between classes but lies between 3–9 amino acid positions proximal of the N terminus of the MHC binding peptide. The 95 percentile confidence limits are slightly different for each panel, from ±0.03–0.05 and are not shown for clarity. Thus the prominent peaks in the graphs are statistically significant but the smaller oscillations of the graphics around zero are not. The corresponding plot for all 11 proteins is shown in Figure S3.4.

To evaluate the relationship between predicted B-cell contact points and MHC-I and MHC-II binding we performed pairwise cross correlation of probability of B-cell epitope binding with the standardized predicted MHC binding of 9-mers and 15-mers. The highest correlation occurs just proximal of the MHC binding index positions. When examined by classes of MHC (Figure 5), we see a characteristic lag period for each of MHC-I Class A, Class B and MHC-II, with remarkable consistency between alleles. Overall, B-cell epitope contact amino acids were found located between 3 and 9 amino acid positions proximal of the N terminus of MHC binding peptides. MHC-I Class B were less closely correlated than MHC-I Class A.

### Correlation of Binding to MHC–I and to MHC-II

**Figure 6:**
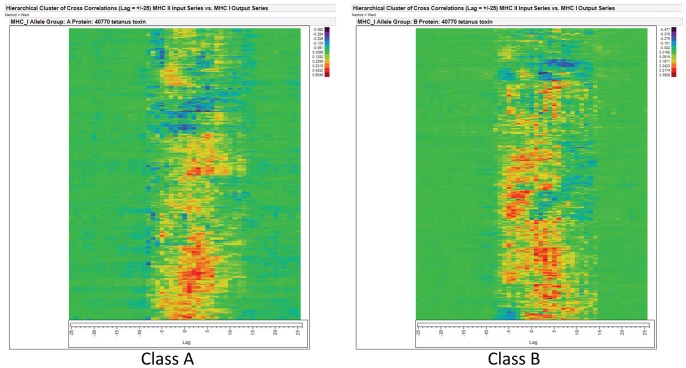
Cross correlation of the position of MHC-I and MHC-II in tetanus toxin. An “all against all” cross correlation was conducted for 28 MHC-II HLA against 20 HLA MHC Class I A (Panel A). This was repeated for 17 alleles of Class I B (Panel B). The vertical line indicates the zero lag position (complete correlation of index position). As both the MHC-I and MHC-II affinities are standardized to zero mean and unit variance, a positive number (red) indicates a strong association between the alleles at that position. A negative number (blue) indicates an anticorrelation between the binding affinities of peptides with an N-terminus at that position. The magnitude and sign of the correlation coefficient for each allele can be determined from the thermometer scale beside the heat diagrams. The corresponding paired plots for all 11 proteins is shown in Figure S3.5.

To evaluate the positional relationship of peptides binding to MHC-I and to MHC-II, we conducted an “all against all” pairwise cross correlation between 28 MHC-II HLA alleles as the input variable and 37 MHC-I HLA alleles (20 Class A and 17 Class B) as the output. Murine alleles were excluded. Figure 6 shows the correlation heat diagrams. There is a strong positional correlation in which a majority of MHC-I binding peptides have their N terminal amino acid approximately 3 amino acids distal of MHC-II binding peptides.

**Figure 7:**
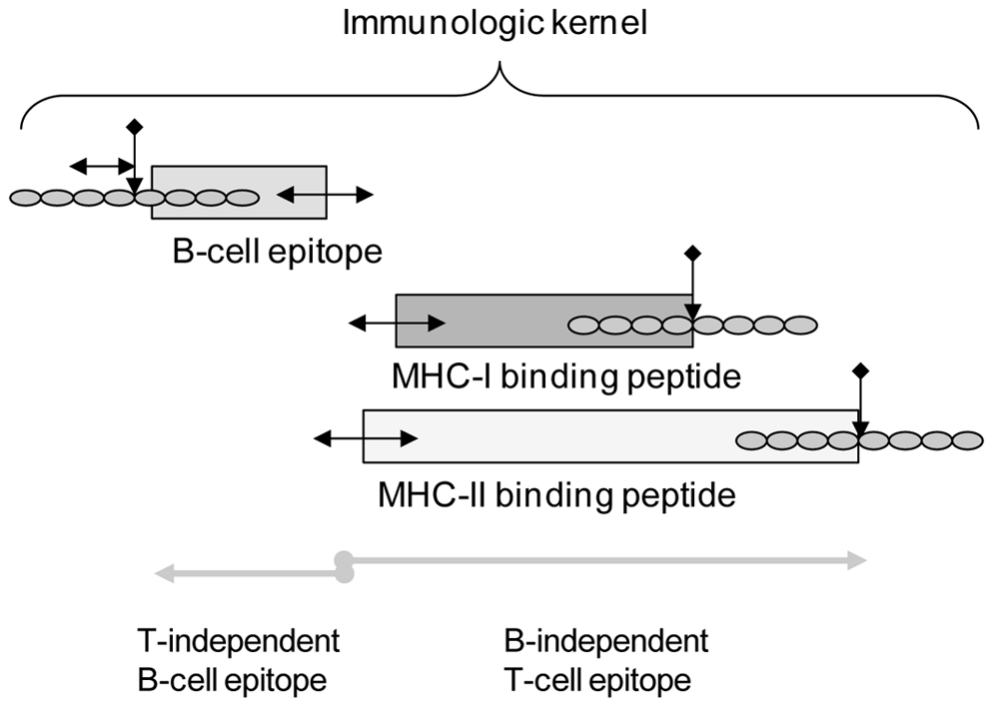
Conceptual model of an immunologic kernel. Relationships of the components are shown based on the cross correlations conducted. Two-headed arrows indicate there will be minor positional differences based on the host MHC alleles. Cathepsin cleavage is a requirement at the C-terminal of the MHC peptides; a high frequency of cathepsin cleavage occurs on the proximal side of the B-cell epitope, but no functional requirement for such cleavage has been demonstrated. Each cleavage site comprises an octomer, with the central P1-P1’ scissile bond indicated by the vertical arrow and the octomer amino acids shown as beads. We have characterized a kernel to comprise both B-cell epitope and T-cell epitope components; as shown T-independent and B-independent epitopes comprise subunits of the whole.

In summary, assembling these relationships our data points to the recurrence of short peptides, of approximately 30 amino acids, bounded proximally and distally by one or more cathepsin cleavage sites and comprising B-cell epitope contact points adjacent to the proximal cathepsin cleavage site and overlapping peptides with a predicted binding with high affinity to MHC-I and MHC-II for one or more alleles with their C termini located at a cathepsin cleavage site and their N termini within about 9 amino acids of a B-cell epitope contact point. Peptides with these patterns occur in clusters, occur repeatedly in protein sequences and have a predominant, specific left-right orientation between the two cleavage delineators. The spatial relationships are summarized in concept in Figure 7. This pattern seen in tetanus toxin is repeated in the other ten proteins we examined and is consistent with our observations of many more proteins.

## Discussion

Our data suggests that the primary amino acid sequences of proteins contain higher order patterns combining sequence elements recognized by both stochastic and genetic components of the immune system. A coordinated, integrated response by the adaptive immune response is thus enabled by a form of symbolic logic, in which multiple signals or conditions are encoded together within short peptides. Each condition can be defined mathematically based on physical properties of amino acids. We refer to these short peptides as “immunologic kernels”. Such immunologic kernels comprise all necessary protein sequence-specific information for the immunological functions of cognition, coordinated activation, and memory recall in a heterozygous individual.

How these primary amino acid sequence elements are processed and presented to the response network is determined by an individual’s immunogenetics. The resultant downstream biochemical signals and cellular effects are a function of which cells take them up, whether as a result of PAMP recognition, B-cell receptor binding, or antibody opsonization, as well as of the cytokine milieu. The many mechanisms extensively documented in the literature address these downstream processes; our focus here is on the ability of the combinatorial primary amino acid sequence elements of a unit peptide to encode the input information. Our predictions show that each individual peptide can accommodate binding peptides for multiple HLA haplotypes. However, each kernel will have peptides of higher or lower binding affinity for specific MHC alleles.

A compact system of immunologic cognition and memory, in which all necessary and sufficient information is contained within a single short peptide may offer a unifying explanation for several observations. An implicit finding is that T-cell help is local; arising for both B-cells and CD8+T-cells from within the same immunologic kernel peptide. This is consistent with the finding of epitope-directed processing [2], [41]. Capture of MHC-binding peptides by B-cell synapse function [9], [10], [42], and the cross presentation by dendritic cells [6] would both be possible by trafficking of a short peptide. Our findings may indicate that long term memory could be encoded within kernel peptides, stored in memory cells, and capable of rapid activation of an integrated response on re-exposure. We observe that MHC-I high affinity peptides are distributed in a more diffuse punctate manner than the clustering seen for MHC-II peptides (example in Figure S1). We have noted, as have others [43], that maximal binding affinity is not always indicative of experimentally reported immunostimulatory epitopes. This may be because a kernel reflects the best compromise of MHC-II and MHC-I binding affinity in close proximity.

While the occurrence of epitopes within immunologic kernels seem to be prevalent as evidenced by the magnitude of the correlation coefficients, exceptions apparently occur in T and B independent epitopes. The spatial relationship of cathepsin cleavage, MHC-I and MHC-II to each other would be maintained in the absence of a B-cell epitope proximally. On the other hand, T-independent B-cell epitopes appear to lack cathepsin B, L and S cleavage sites as well as high affinity MHC binding (See Figure S5).

Any antigen presenting cell may have multiple cathepsins active; the relative role of which will vary by cell type and cytokine milieu [26], [30], [31], [44]. Cathepsin L and S are similar in action in defining the C terminus of a MHC binding peptide. Our analysis shows that in tetanus toxin the mean cleavage distance by cathepsin L is 2.85 amino acids and for cathepsin S is 4.67 amino acids. We also show cleavage has a low probability within an “exclusion zone” of 5 amino acids either side of a cleavage site. Hence, peptides less than 8–10 amino acids are an unlikely result from the action of these two cathepsins, but could arise though other endopeptidase action. In the event that smaller fragments were generated they would be unlikely to bind competitively compared to a peptide capable of occupying the entire binding groove.

A number of new questions arise. While variable lengths of MHC-I binding peptides are expected, we were surprised to find the prediction of MHC-I initiation sites located 10 amino acids from the cathepsin L or S cleavage site, rather than a consensus nonamer which is also the basis of our neural net-training sets. A number of predicted high affinity peptides are found with a nine amino acid length but the highest cross correlation is for ten amino acid peptides. Interestingly, the predicted cleavage by the 20S proteasome produces 9-mers that are preferred by some MHC class I alleles (Figure S4). If the negative correlations we show between cleavages at ±5 from a primary cleavage point are relevant to the peptide excision process, then 10 amino acids would be a next (proximal or distal) potential site of an initial cathepsin cleavage event. Similarly the 16 amino acid offset for MHC-II and the second correlation at a 5–6 amino acid position lag suggests the action of sequential cleavage sites. B-cell epitopes are positioned proximal of MHC binding peptides. This finding is consistent with the physical property measurements of Melton and Landry [45], [46] who observed CD4^+^epitopes located in the same orientation we observe, on the flanks of flexible regions of protein which would be apt to contain B-cell epitopes and adjacent to proteolytic cleavage sites. Moss *et al* also showed a left right B-cell epitope T_H_ pattern experimentally [11]. The repeated patterns are seen in proteins of widely varying lengths; the signals are stronger in longer proteins because there is more chance for pattern reinforcement.

The evidence we present suggests that linear peptides contain sufficient information to mobilize all components of an adaptive response. However, three dimensional B-cell epitopes are well documented [47]; do these comprise multiplicatively reinforcing kernels or is crossover of help between kernels a factor? Is all T-cell help local? That would be consistent with experimental findings with synthetic peptides [14]. Natural experiments of immune escape tend to support the concept that local help may at least be the most important [23]. Asparagine endopeptidase clearly plays a role in release of longer peptides as a prerequisite to MHC-II binding [11]. It is unclear whether endopeptidases other than cathepsin L and S can deliver the shorter “kernel” peptides, perhaps depending on cell type [48], [49]. At this time there are no training sets to enable us to predict cleavage for other endosomal peptidases. There may also be endosomal carboxypeptidase trimming of the 10-mers produced by cathepsin S or L down to a 9-mer. We note that as cathepsin S may be upregulated by interferon [44], an interferon induced bias towards cathepsin S could potentially slightly increase the average size of peptides released, as cathepsin S has a different cleavage frequency from cathepsin L. The distribution of cathepsin L and S is cell type dependent [30], [31]. We can speculate on what evolutionary advantage an immunologic kernel offers, given that the information will be read in multiple frames by different HLA alleles in a heterozygous individual. Intuitively, close spatially associated cleavage and binding events would seem to have a higher probability of being repeated in the memory phase of the adaptive response. Furthermore, they would have a higher probability of being conserved in alternatively spliced isoforms, now thought to be generated by all multi-exonic genes [50], enabling continued self recognition. The need for multiple combinatorial signals sets a higher criterion for initiation and recall of an immune response.

The spatial integration of facets of the immune response we describe comprises features consistent with many published descriptions of components of the immune response. However, researchers tend to specialize in studies of one arm of the immune response. Those who approach mapping of epitopes with short overlapping peptides may overlook the need for integrity of the cleavage site octomer either side of the cathepsin cleavage site. Conversely mapping of epitopes using extended polypeptides lacks the precision to demonstrate the relationships. By using bioinformatic processes we have taken a higher level view of immunologic patterns to see features invisible at the bench experimental level. As a result we offer a unifying hypothesis for the integrated function of the adaptive immune system which must now be further tested at the bench level.

## Methods

All data analysis was performed with scripts written for and implemented within JMP® v10 (SAS Institute, Cary, North Carolina). MHC binding affinities and B-cell epitope contact points were predicted using techniques previously described and validated [21]–[23]. Probability of peptide cleavage likewise predicted based on discriminant equation ensembles derived by use of PCAA in conjunction with a probabilistic neural network for all possible amino acids in a scissile bond (P1-P1’) pair (see File S1). The cleavage site octomer primary sequences used to train the neural network in JMP® v10 were derived from large published datasets [24], [25]. The primary amino acid sequences of the proteins in the present study were vector encoded as the first three PCAA physical properties and resultant vectors used as input to discriminant equation ensembles to derive a predicted cleavage probability. A BEPI probability score based on B-cell epitope contact points were predicted using amino acid principal components with a neural network (PCAA-NN) based on published training sets [39]. On test datasets the Pearson correlation coefficient between the PCAA-NN and BepiPred (http://www.cbs.dtu.dk/services/BepiPred/) was 0.93.

To produce normally distributed data required for reliable statistical analysis predicted binding affinities (as the natural logarithm) of all peptides indexed by single amino acids were standardized to zero mean and unit variance using a bounded Johnson (Sb) distribution [51]. Standardization was done individually for each allele within each protein. Thus, all comparisons within and between alleles assumes the data are normally distributed. Hierarchical clustering of the metrics was done by the minimum variance method of Ward [52]. Time series analysis was applied to the numerical-vector-encoded sequences data using the Time Series modeling platform in JMP® v10. The white noise test for the presence of periodic patterns in the sequence data used Fisher’s Kappa statistic that tests the null hypothesis that the values in the series are drawn from a normal distribution with variance 1 against the alternative hypothesis that the series has some periodic component [53]. Kappa is the ratio of the maximum value of the periodogram and its average value. The horizontal lines in the cross correlation plots are drawn at the point of the 95-percentile confidence limit. The JMP® application reports the standard error of the cross-correlation coefficient and this was multiplied by two in order to provide the reader an overall sense of the level of statistical significance of the numerical values.

## Supporting Information

Figure S1 **Detailed MHC affinity and B cell epitope mapping of tetanus toxin.**
(PDF)

Figure S2 **Immunologically relevant combinatorial patterns of amino acids in primary amino acid sequences.**
(PDF)

Figures S3 **Cross-correlation analyses derived from all 11 proteins in** **Table 1** **.**
(PDF)

Figure S4 **Cross-correlation of MHC-I predicted binding affinity relative to Netchop 20S proteasome cleavage of tetanus toxin.**
(PDF)

Figure S5 **Detailed MHC affinity and B-cell epitope mapping of** ***Staphylococcus aureus*** **iron sensitive determinant B illustrating features of T-independent epitopes.**
(PDF)

File S1 **Summary of cathepsin cleavage prediction methodology.**
(PDF)

Table S1 **Excel spread sheets of predicted affinity and cleavage for tetanus toxin.**
(XLXS)
