# Supplementary material for: Recognition of Higher Order Patterns in Proteins: Immunologic Kernels
Source: PLoS One. 2013 Jul 29;8(7):e70115. doi: 10.1371/journal.pone.0070115 (PMC3726486; doi:10.1371/journal.pone.0070115)

Figure S1: Detailed MHC affinity and B cell epitope mapping of tetanus toxin (gi: 40770)

A & B: Hierarchical cluster of binding of peptides in tetanus toxin to individual MHC alleles (Y axes). On the X axis 9-mer and 15-mer peptides respectively are indexed to their N terminal positions. Color index shows binding affinity in standard deviations units (blue high affinity). Shows HLA which react similarly, as well as variability in binding affinity by peptide.

C. Population phenotype: Predicted MHC-I (red line), MHC-II (blue line) binding, and probability of B-cell binding (orange lines) for each peptide, arrayed N-C, for a permuted population comprising 66 human MHCs (MHC-I Class A Class B and MHC-II DR only) Ribbons (Red=MHC-I, Blue-MHC-II) indicate the top 25% affinity binding. Orange bars indicate high probability B-cell binding.

To obtain permuted averages all possible allelic pairs in heterozygous and homozygous crosses are compared and the highest affinity binder of each pair averaged. Methodology as described in: Bremel RD, Homan EJ: An integrated approach to epitope analysis II: A system for proteomic-scale prediction of immunological characteristics. *Immunome Research* 2010, 6:8.

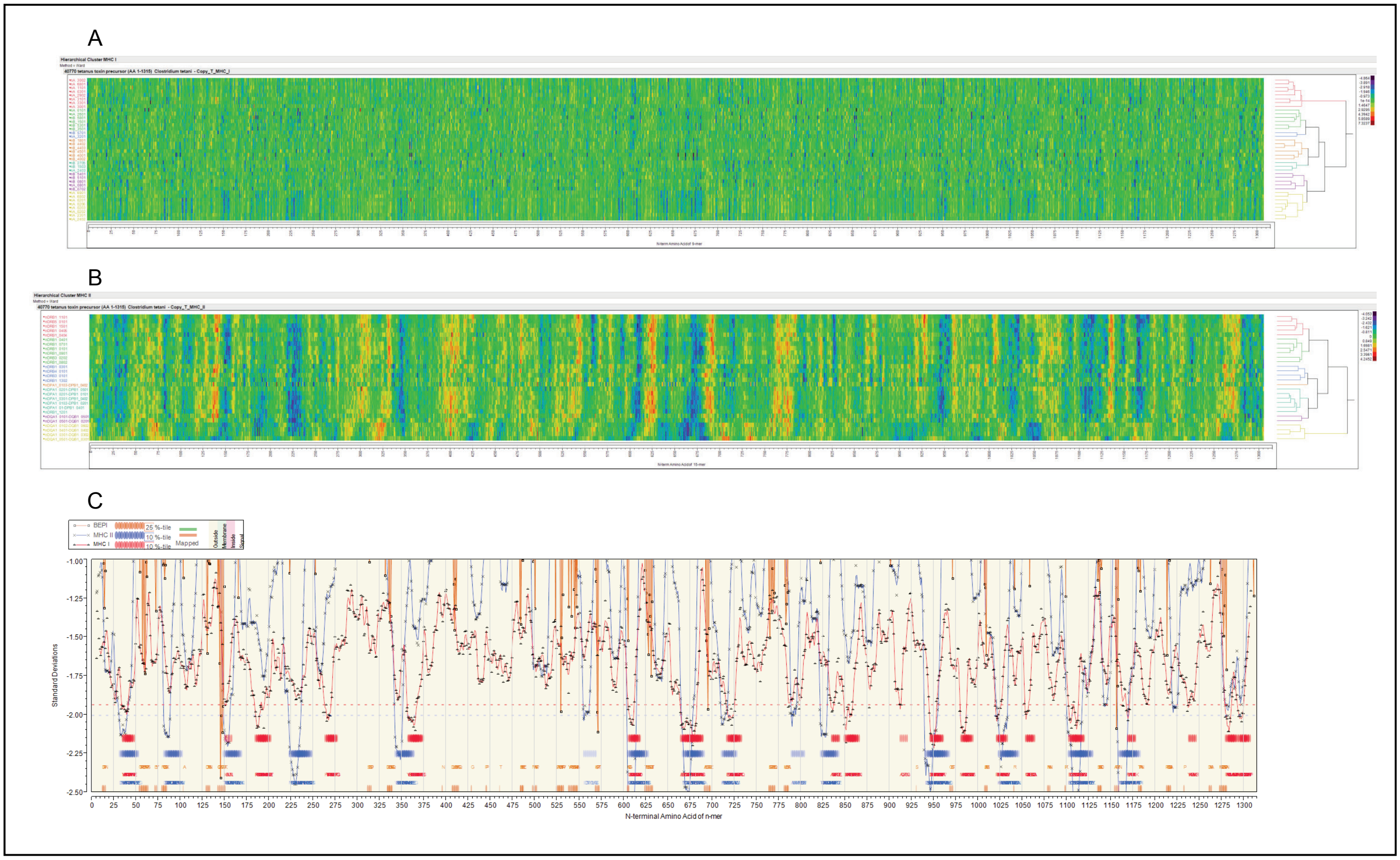

Supplement: Figure S1 — Detailed MHC affinity and B cell epitope mapping of tetanus toxin. (PDF) [file pone.0070115.s001.pdf]
