# Supplementary material for: Recognition of Higher Order Patterns in Proteins: Immunologic Kernels
Source: PLoS One. 2013 Jul 29;8(7):e70115. doi: 10.1371/journal.pone.0070115 (PMC3726486; doi:10.1371/journal.pone.0070115)

Figure S2: Immunologically relevant combinatorial patterns of amino acids in primary amino acid sequences.

Spectral density periodograms of amino acid sequence metrics with accompanying statistical tests for estimating whether the pattern is different from white noise. All sequence metrics were standardized to zero mean and unit variance prior to the analysis. Panel A: B-cell contact point probability. Panels B,C,D,E & F: representative patterns for several MHC-II alleles; Panels G,H, I &J: representative patterns for several MHC-I alleles; Panel K: Cathepsin S; Panel L: Cathepsin L. The B-cell contact point pattern as well as those for the binding affinities of MHC-II alleles are all statistically different from white noise. However, the MHC-I binding affinities as well as the cleavage probabilities for cathepsin S and cathepsin L all indistinguishable from random numbers. Note the differences in scale and the values of the Kappa statistic. Fisher's Kappa statistic tests the null hypothesis that the values in the series are drawn from a normal distribution with variance 1 against the alternative hypothesis that the series has some periodic component. Kappa is the ratio of the maximum value of the periodogram and its average value.

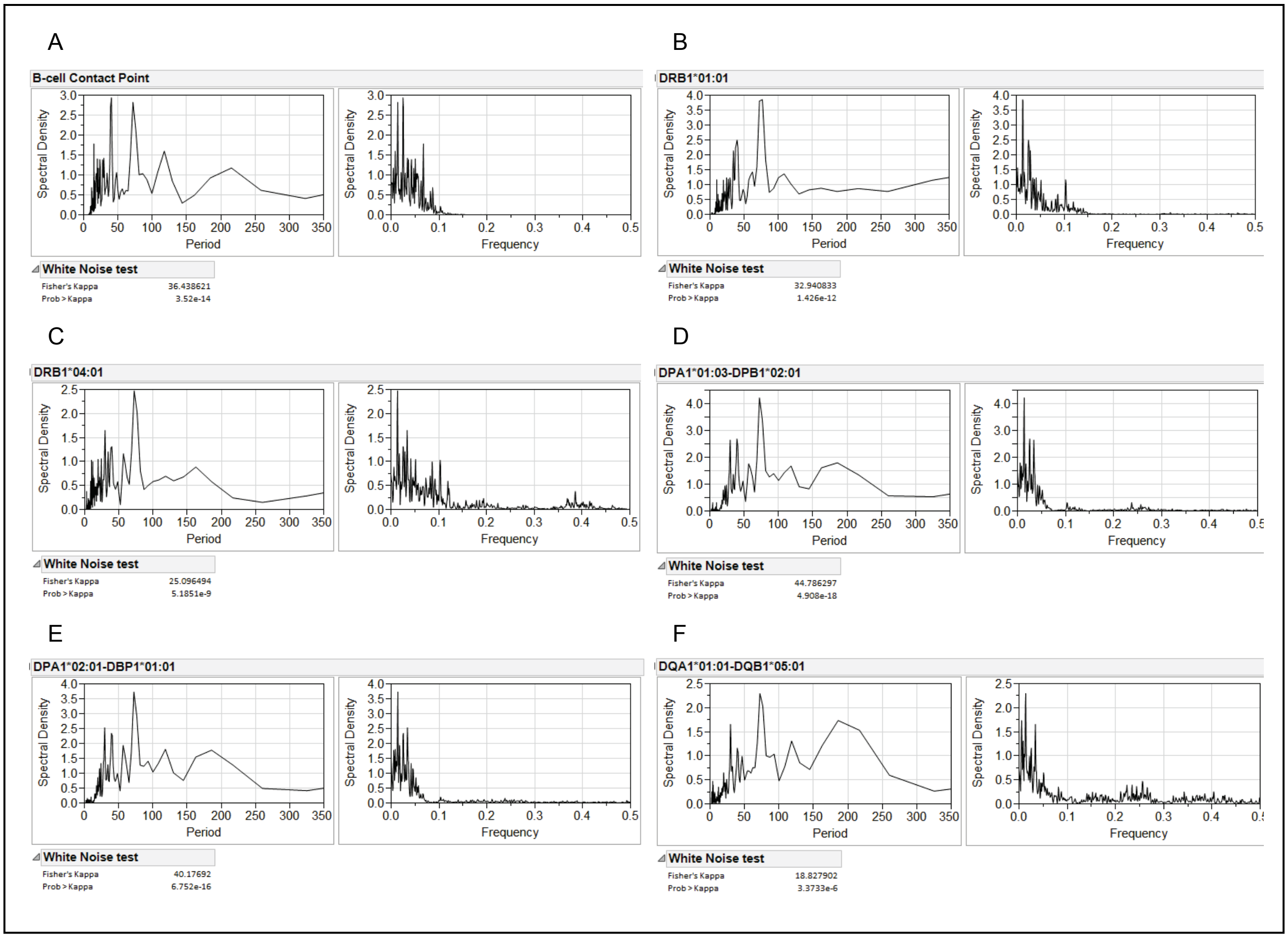

Figure S2 continued.

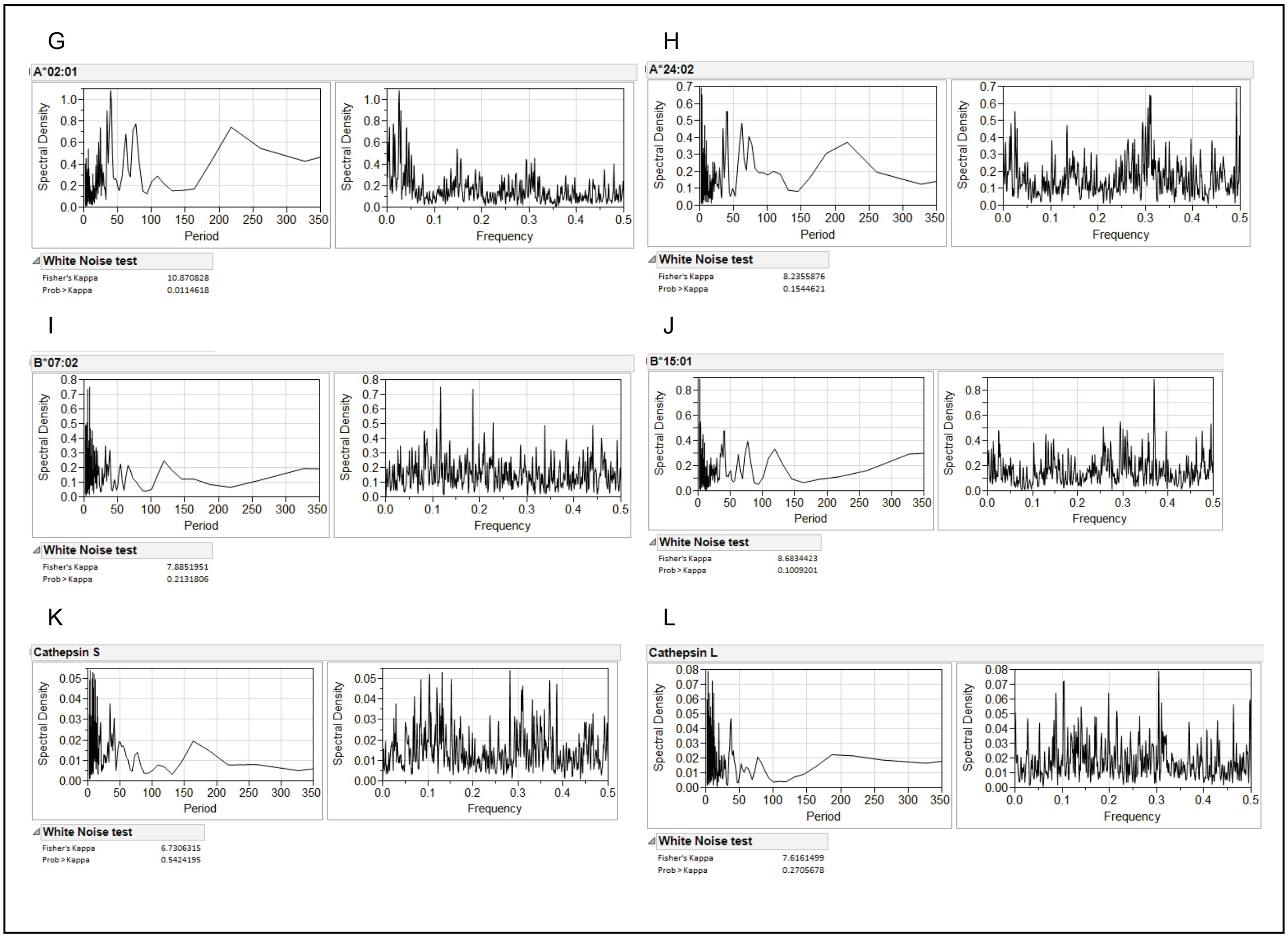

Supplement: Figure S2 — Immunologically relevant combinatorial patterns of amino acids in primary amino acid sequences. (PDF) [file pone.0070115.s002.pdf]
