# Supplementary material for: Recognition of Higher Order Patterns in Proteins: Immunologic Kernels
Source: PLoS One. 2013 Jul 29;8(7):e70115. doi: 10.1371/journal.pone.0070115 (PMC3726486; doi:10.1371/journal.pone.0070115)
Supplement: Figures S3 — Cross-correlation analyses derived from all 11 proteins in Table 1 . (PDF) [file pone.0070115.s003.pdf]

Figures S3.1-S3.5. These comprise figures derived from all 11 proteins in Table 1.

For reference the proteins in Table 1 are listed below:

Mumps hemagglutinin_neuraminidase Jeryl Lynn Minor, gi19070176
Staph. aureus Cell surface receptor IsdB, gi 19528514
Staph. aureus Cell surface receptor IsdH, gi 19528514
Foot-and-mouth disease virus P1 polyprotein, gi 311701499
Diphtheria toxin, gi 38232848
Tetanus toxin precursor, gi 40770
Human coagulation factor VIII isoform a, gi 4503647
Brucella melitensis polynucleotide phosphorylase_polyadenylase, gi17988244
Brucella melitensis methionine sulfoxide reductase B, gi17989164
Arachis hypogaea Ara h 6 allergen, gi57118278
Arachis hypogaea LTP isoallergen, gi1161087230

S3.1 Cleavage site specificity overlap and cross-correlation between predicted cathepsin B, cathepsin L and cathepsin S cleavage positions in the eleven proteins of interest.

S3.2 Cross-correlation of the predicted MHC binding peptide N terminus with cathepsin L cleavage for 11 proteins.

S3.3 Cross-correlation coefficients of B- cell epitope contacts with cathepsin L cleavage probability in 11 proteins

S3.4 Cross-correlation coefficients of MHC binding peptide N terminus and B-cell epitope contacts in 11 proteins.

S3.5 Hierarchical clustered heat diagrams of cross-correlation coefficients of index positions of 9-mer peptides based on predicted MHC-I affinity and index positions of 15-mer peptides based on predicted MHC-II affinity

Figure S3.1: **Cleavage site specificity overlap and cross-correlation between predicted cathepsin B, cathepsin L and cathepsin S cleavage positions in the eleven proteins of interest.**

Predicted cathepsin B, L, and S cleavage sites in the eleven proteins in Table 1 were tabulated and cross correlated. **A:** Venn diagram of redundancy of predicted cathepsin cleavage at particular P1-P1' positions in a cleavage site octomers. A cleavage probability threshold was set at 0.5. The circles are proportional to the total numbers of cleavages by the particular peptidase and the numbers in the overlaps indicates the commonality in cleavage site specificity. **B:** Cross-correlation of cleavage predictions of cathepsin L and cathepsin S. This shows that the two cathepsins tend to cut at the same place. The cleavage patterns of proteins often appears as a cluster of 1-3 cleavages in specific regions. This concept is reinforced by the cleavage correlations at ± 1. The negative peaks at ± 4-5 can be interpreted as meaning that there is unlikely to be a cleavage immediately upstream or downstream by 4 or 5 amino acids. **C:** Cross correlation of cathepsin B with cathepsin S. **D:** Cross correlation fo cathepsin B with cathepsin L. This shows that cathepsin B patterns are quite different from the other two but that cathepsin B has a tendency to cleave in the same region. Each error bar = 1 standard deviation from the mean. The 95th percentile confidence limits are different for each protein and for each panel but range from ±0.02 – 0.05 and are not shown for clarity. Thus the prominent peaks in the graphs are highly statistically significant but the smaller oscillations of the graphics around zero are not. See also Figure 1 in the main text.

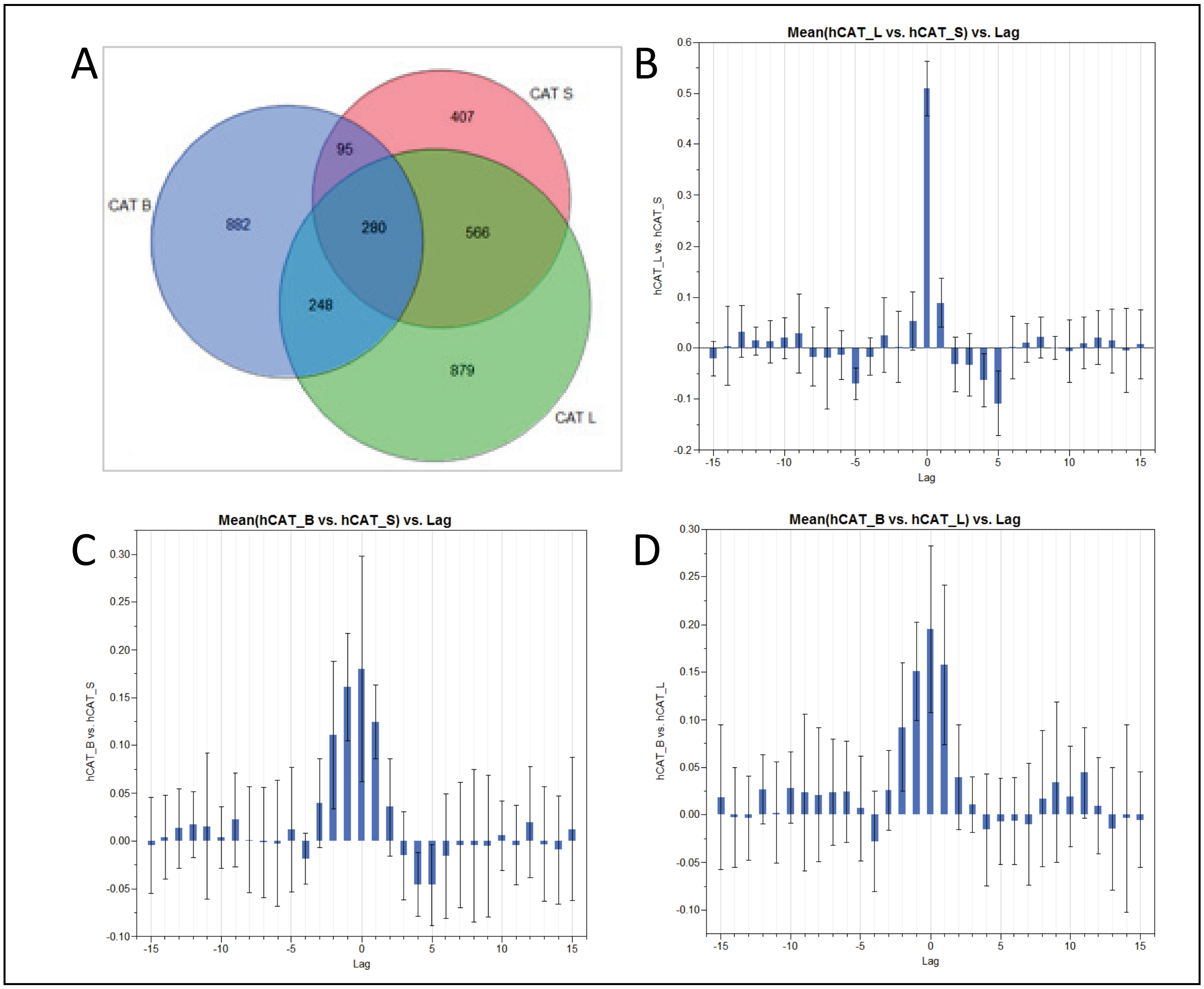

Figure S3.2. Cross-correlation of the predicted MHC binding peptide N terminus with cathepsin L cleavage for 11 proteins.

Panel A: MHC-I; Panel B:MHC-II. The vertical line at position 3 represents the P1P1' scissile bond. Bars either side of the mean indicate 10/90 percentile, boxes 25/75 percentile. While these figures show a wide variation it should be realized that the graphs comprise a composite of the results for all the indicated alleles for 8192 amino acids in eleven different proteins. Some of the individual alleles have peak correlations ranging from 0.5-0.8 and some have different signs at the different locations. The fact that there is a pattern is quite remarkable. The 95 percentile confidence limits are different for each protein and for each panel but range from ±0.02 – 0.05 and are not shown for clarity. Thus the prominent peaks in the graphs are highly statistically significant but the smaller oscillations of the graphics around zero are not. See also Figure 3 in the main text.

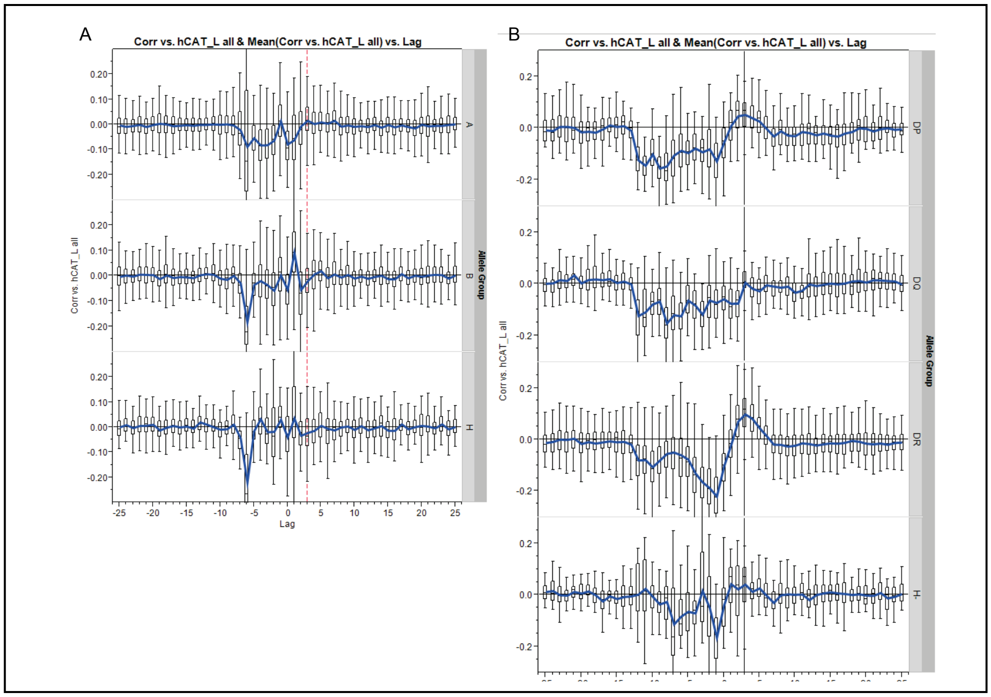

Figure S3.3. Cross-correlation coefficients of B- cell epitope contacts with cathepsin L cleavage probability in 11 proteins

Vertical red line marks the B-cell contact probability, hence cathepsin cleavage is unlikely within 3 amino acids distal or 6 amino acids proximal of the center of B cell epitope. Bars either side of the mean indicate 10/90 percentile, boxes 25/75 percentile. This is the composite result for 8192 amino acid relationships in 11 different proteins. The 95th percentile confidence limits are approximately ±0.03 and are not shown for clarity.

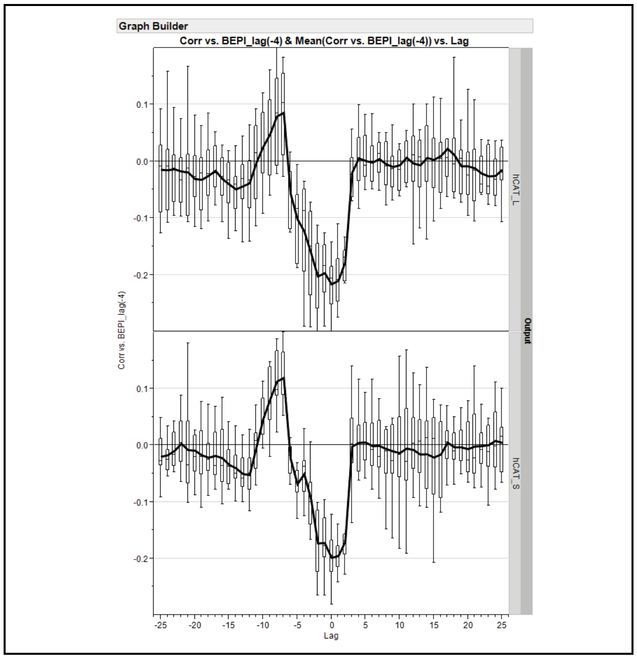

Figure S3.4: Cross-correlation coefficients of MHC binding peptide N terminus and B-cell epitope contacts in 11 proteins.

Panel A: MHC-I binding peptides, Y axis correlation coefficients for Class A, Class B, and Murine (H). Panel B: MHC-II binding peptides Y axis: correlation coefficients for DP, DQ, DR and Murine (H). Bars either side of the mean indicate 10/90 percentile, boxes 25/75 percentile On average B-cell epitope contacts are centered proximal of the MHC N terminus by 3-9 amino acids. This is the composite result for 8192 amino acid relationships in 11 different proteins. The 95th percentile confidence limits are approximately range from ±0.015 -0.03 and are not shown for clarity.

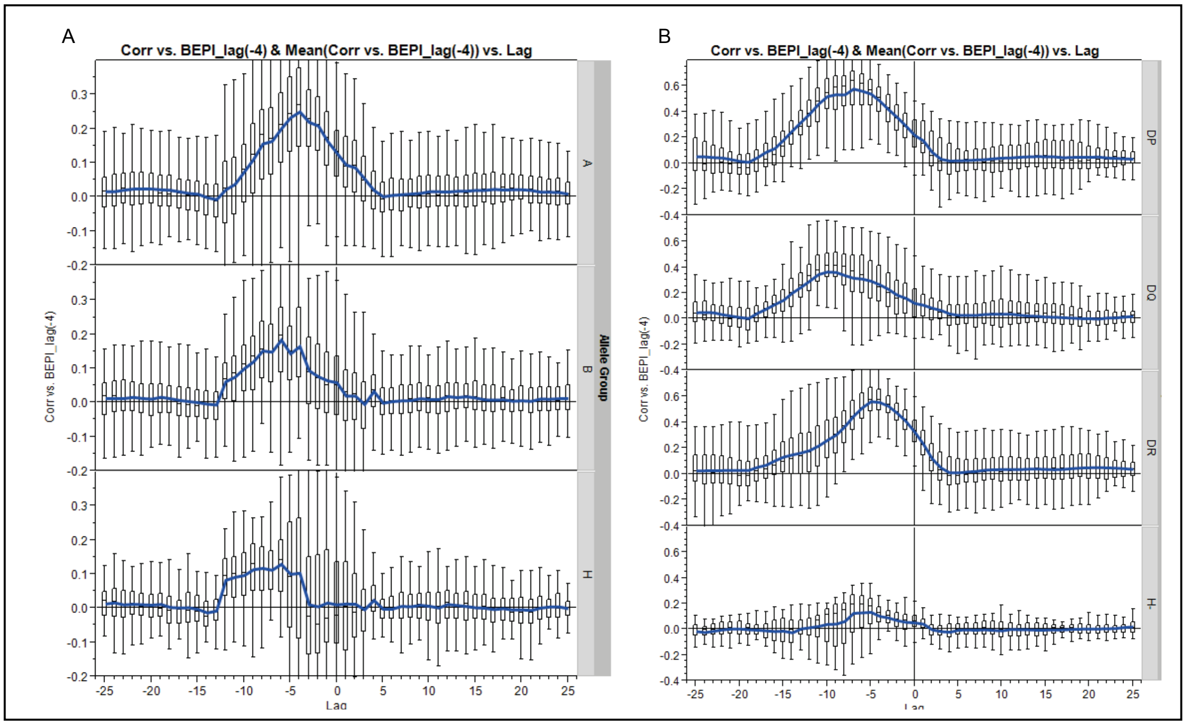

Figure S3.5. Hierarchical clustered heat diagrams of cross-correlation coefficients of index positions of 9-mer peptides based on predicted MHC-I affinity and index positions of 15-mer peptides based on predicted MHC-II affinity

All against all correlations were conducted taking 28 MHC II alleles as input and output as 20 Class A or 17 Class B MHC-I alleles. Two panels are thus shown for each protein. In contrast to the prior figures these patterns are for a composite of all peptides within each of the specific proteins. These patterns patterns show close correlation of predicted high affinity binding, with lag positions of the MHC-I index positions lying distal of the MHC-II positions. These patterns are simply to show the general relative phase relationships in the different proteins. The magnitudes of some of the correlations are quite high and can be seen in the zoomable thermometers associated with each panel. Overall the general allelic patterns for different pairs are similar in each protein.

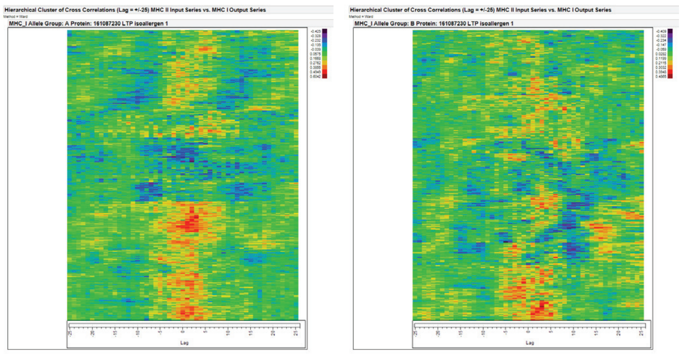

Arachis hypogaea LTP isoallergen, gi1161087230

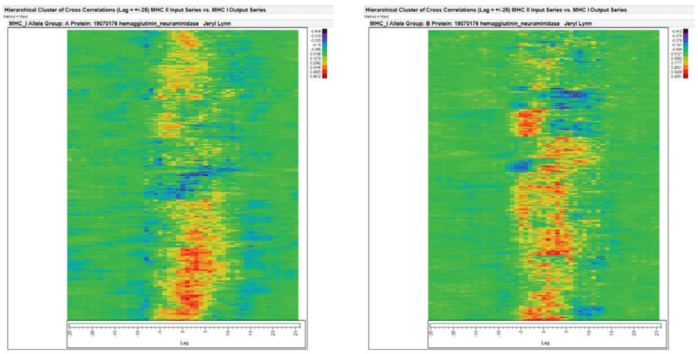

Mumps hemagglutinin_neuraminidase Jeryl Lynn Minor, gi19070176

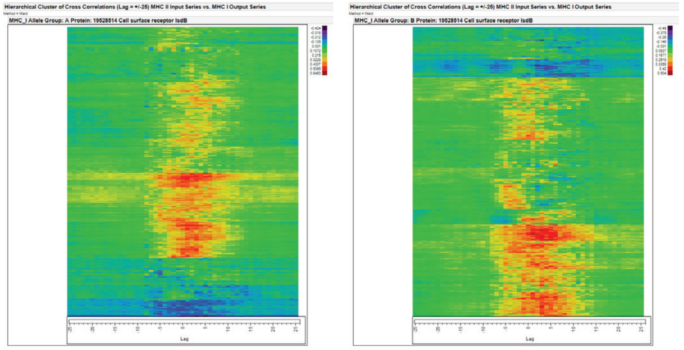

Staph. aureus Cell surface receptor IsdB, gi 19528514

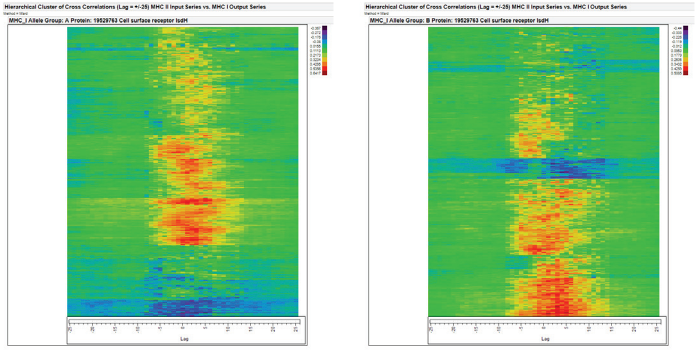

Staph. aureus Cell surface receptor IsdH, gi 19528514

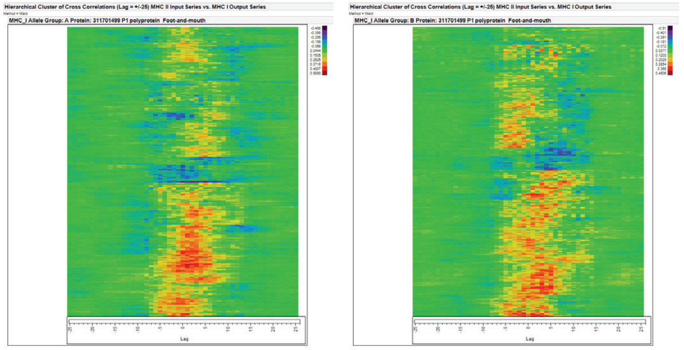

Foot-and-mouth disease virus P1 polyprotein, gi 311701

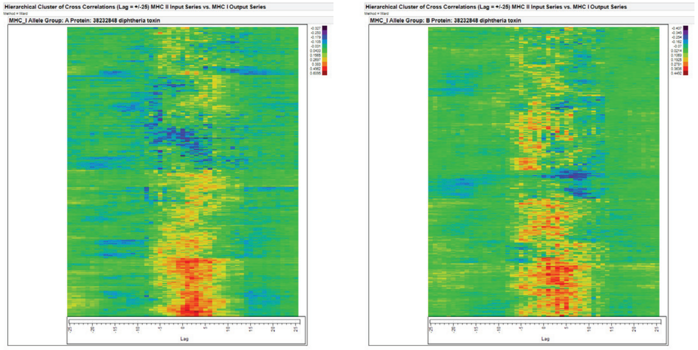

Diphtheria toxin, gi 38232848

Figure S3.5. continued

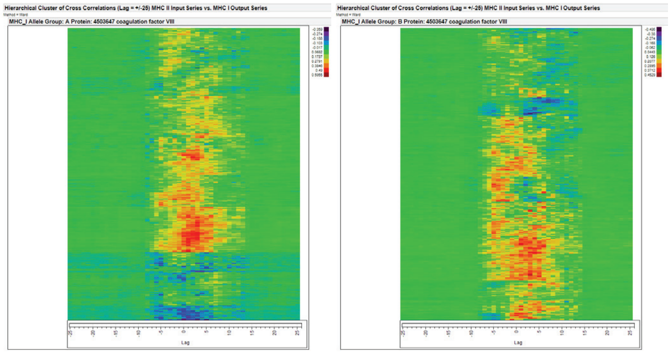

Human coagulation factor VIII isoform a, gi 4503647

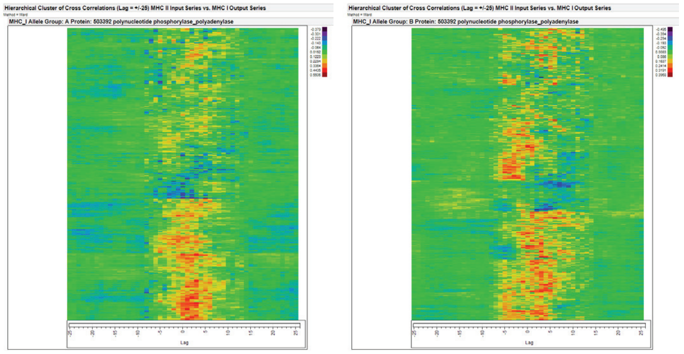

B.melitensis polynucleotide phosphorylase_polyadenylase, gi1798824

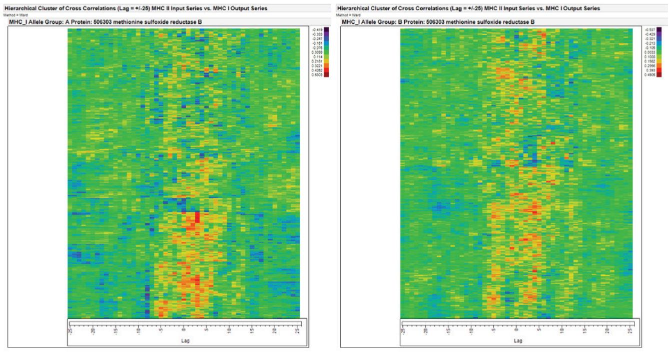

B. melitensis methionine sulfoxide reductase B, gi17989164

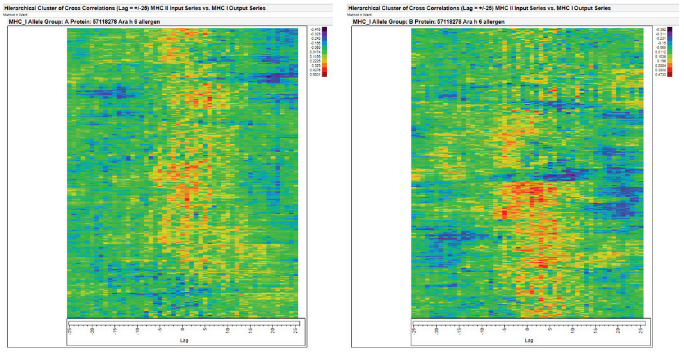

Arachis hypogaea Ara h 6 allergen, gi57118278
