# Supplementary material for: Recognition of Higher Order Patterns in Proteins: Immunologic Kernels
Source: PLoS One. 2013 Jul 29;8(7):e70115. doi: 10.1371/journal.pone.0070115 (PMC3726486; doi:10.1371/journal.pone.0070115)
Supplement: Figure S4 — Cross-correlation of MHC-I predicted binding affinity relative to Netchop 20S proteasome cleavage of tetanus toxin. (PDF) [file pone.0070115.s004.pdf]

Figure S4: Cross-correlation of MHC-I predicted binding affinity relative to Netchop 20S proteasome cleavage of tetanus toxin

The sequence for tetanus toxoid (gi 40770) was submitted to NetChop 3.1 webserver[1] (http://www.cbs.dtu.dk/services/NetChop/)and the cleavage predictions for the 20S proteasomal subunit were retrieved. The output was cross-correlated against standardized individual MHC-I binding affinities indexed to the N-terminal amino acid of the binding 9-mer peptide. The scissile bond cleavage position is indicated using the standard peptidase notation.

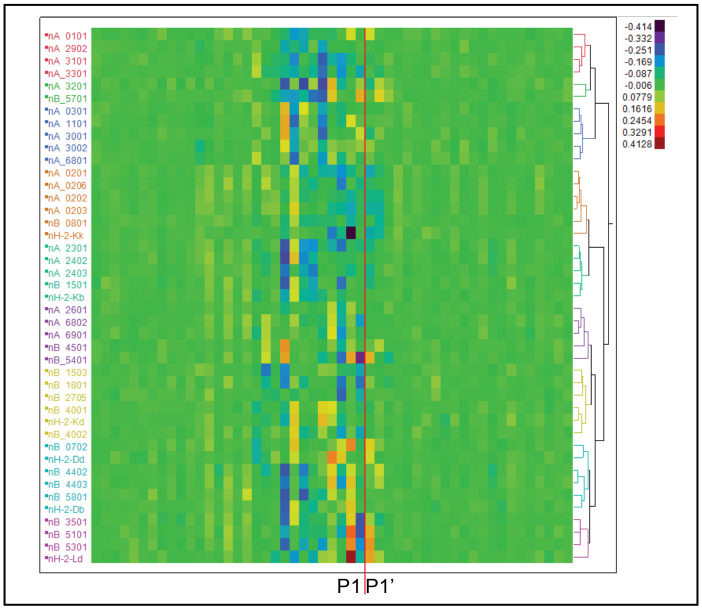

1 Nielsen, M., Lundegaard, C., Lund, O. & Kesmir, C. The role of the proteasome in generating cytotoxic T-cell epitopes: insights obtained from improved predictions of proteasomal cleavage. *Immunogenetics* **57**, 33-41, doi:10.1007/s00251-005-0781-7 (2005).
