# Supplementary material for: Recognition of Higher Order Patterns in Proteins: Immunologic Kernels
Source: PLoS One. 2013 Jul 29;8(7):e70115. doi: 10.1371/journal.pone.0070115 (PMC3726486; doi:10.1371/journal.pone.0070115)

Figure S5: Detailed MHC affinity and B cell epitope mapping of Staphylococcus aureus iron sensitive determinant B illustrating features of T-independent epitopes.

Panel A: Population permuted plot of Staph aureus IsdB, gi 19528514

Predicted MHC-I (red line), MHC-II (blue line) binding, and probability of B-cell binding (orange lines) for each peptide, arrayed N-C, for a permuted population comprising 66 human MHCs (MHC-I Class A Class B and MHC-II DR only) Ribbons (Red=MHC-I, Blue-MHC-II) indicate the top 25% affinity binding. Orange bars indicate high probability B-cell binding. Background shading shows membrane (green) extramembrane (yellow), intramembrane (pink). Areas with many T-independent B-cell epitopes are seen at amino acid positions 465-625 and to a lesser degree at positions 40-100.

To obtain permuted averages all possible allelic pairs in heterozygous and homozygous crosses are compared and the highest affinity binder of each pair averaged. Methodology as described in: Bremel RD, Homan EJ: An integrated approach to epitope analysis II: A system for proteomic-scale prediction of immunological characteristics. *Immunome Research* 2010, 6:8.

Panel B. Probability of cathepsin cleavage for the same protein by murine cathepsins (mCAT) and human cathepsins (hCAT). It can be seen that there is a relatively lower level of cathepsin activity predicted in the sequences with T-independent B cell epitopes seen in Panel A.

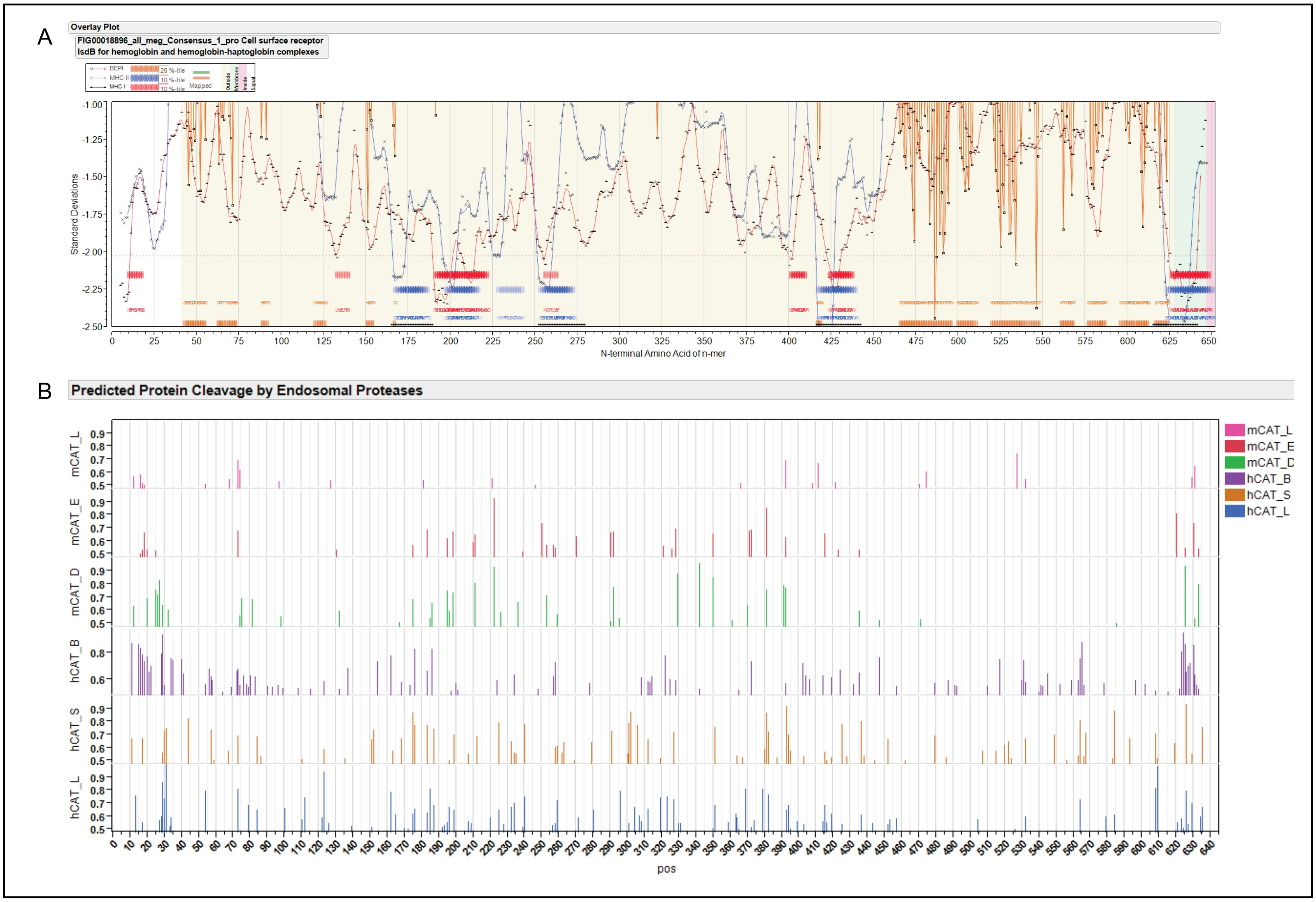

Supplement: Figure S5 — Detailed MHC affinity and B-cell epitope mapping of Staphylococcus aureus iron sensitive determinant B illustrating features of T-independent epitopes. (PDF) [file pone.0070115.s005.pdf]
