# Supplementary material for: Recognition of Higher Order Patterns in Proteins: Immunologic Kernels
Source: PLoS One. 2013 Jul 29;8(7):e70115. doi: 10.1371/journal.pone.0070115 (PMC3726486; doi:10.1371/journal.pone.0070115)
Supplement: File S1 — Summary of cathepsin cleavage prediction methodology. (PDF) [file pone.0070115.s006.pdf]

File S1

Summary of cathepsin cleavage prediction methodology.

Several new proteomic techniques have recently been developed that identify newly created cleavage sites through isotopic or other labeling of the residues flanking the cleavage site, and mass spectrometry *[1-5]*. These cleavage site labelling (CSL) techniques can characterize in a single experiment hundreds of times as many cleavage events as previously catalogued for a peptidase.

The active site of peptidases can be conceptualized as an enzyme binding pocket with 8 contiguous topographic subsites comprising the cleavage site octomer (CSO) are consecutively numbered outward from the cleavage site between P1 and P1' as P4-P3-P2-P1|P1'-P2'-P3'-P4'. The corresponding binding sites on the enzyme are numbered S4…S4'. The similarity between the octomer binding pocket of a peptidase and the binding pocket of an MHC molecule is obvious and suggested that a similar process might be developed for predicting peptidase cleavage probabilities instead of MHC binding affinities. The mass of data produced by CSL techniques, and the randomness of the peptide sequences, provided the input for developing training sets to which we applied principal component analysis of amino acid (PCAA) physical properties in a similar approach to our recent analysis of MHC binding affinity prediction [6,7].

We showed that PCAA can be used to develop classifiers for prediction of peptidase cleavage sites using the much larger datasets produced by the CSL proteomic processes and provided in the Supplemental materials of three recent publications [8-10]. We used a probabilistic neural network perceptron that is essentially a non-linear PLS regression [11] provided in a widely used statistical program (JMP®) to develop the prediction equations. As with our earlier work with MHC binding predictions, we use a perceptron with mathematical symmetry to the biological subject, in this case the peptidase binding pocket. We showed that the process generates accurate predictions of cleavages for each of the peptidases studied.

Non-redundant octomer datasets were derived from the CSL datasets, indexed by single amino acid displacement throughout the protein, and used for training the neural net. Non-redundant sets consisting of a single representatives of a particular CSO were created because the presence of multiple copies of a particular cleaved peptide could be due either to parent protein abundance in the protein mixture used or

because of the cleavability by a particular peptidase. Since all of the proteins in these experimental sets were exposed to the peptidase for extended periods of time and only a relatively small number of sites were cleaved, the non-cleaved peptides were assumed to be true negatives for classifier training purposes.

The peptidase prediction output is a binary categorical variable (cleave/no-cleave) rather than a continuous real number as in MHC binding affinity prediction (i.e. natural logarithm ic_{50}). The perceptron is the topological description of the underlying mathematical equation lattice comprising a neural network. For this purpose a relatively simple perceptron was used having a single input layer which comprises the amino acid principal component vectors, a single layer of hidden nodes and a single binary output that is the predicted probability of cleavage coded as a zero or one. The number of hidden nodes was fixed at eight, providing symmetry between the mathematics of the perceptron and the conceptual underlying enzyme binding pocket.

The PCAA were derived by eigen decomposition of the correlation matrix of 31 different studies as described previously [7]. Use of the correlation matrix as a foundation makes it possible to combine the results of a wide variety of studies with different scoring metrics to create a composite set of vectors that are mutually orthogonal (i.e. uncorrelated), zero-centered, and appropriately weighted for their relative contributions. The peptidase cleavage datasets provided by the CSL studies have 500 to 3000 cleaved peptides that exceeded the detection limit of mass spectrometry for each experimental conditions in a total of up to approx 800 identified proteins. For cathepsin L training sets resulting from three different experimental conditions were used and for cathpesin S training sets resulting from two different experimental conditions were used. The conditions of cleavage under which the experiments that produced the training sets were done at pH 6 (cathepsin L and cathepsin S) and pH7.5 (cathepsin S) to represent the range pH conditions found in the endosome compartments at different stages of antigen processing.

We used an approach shown to produce robust results for diverse situations to “optimize locally and apply globally” by creating small, balanced subsets (ensembles) for training (bootstrap aggregation, “bagging”) and validation of the classifiers and then using the resulting predictors for larger datasets. A 5 k-fold cross validation was performed 5 times, each time starting with a different seed for the random number generator.

Ensembles of 25 discriminant equations were produced for each amino acid found at the P1 and P1' position of the CSO. As nearly every amino acid, of the 20 possible, was found at each of those positions up to nearly 1000 total discriminant equations were produced for each experimental set. Each of the members of the ensemble comprises a randomly selected, independent predictor of the probability of the cleavage of a peptide at a certain P1-P1' pair based on the combinatorial amino acids in the flanking positions. These equations performed very well with a true positive rate of approximately 90% and and a false positive rate of about 10%. Predictions varied for different amino acids and were more reliable for some than for others. Peptides that contained multiple identical amino acids result had in the lowest overall performance (75-82% True Positive prediction accuracy). The probabilities from the multiple different training sets were combined and the maximum probability of the group of predictions was used as the metric (cathepsin L = six total predictions, P1 and P1' for 3 different experimental conditions; cathepsin S = four total predictions, P1 and P1' for 2 experimental conditions).

Technical Appendix Figure 1.1

Sensitivity and specificity patterns for human cathepsin S at pH 7.5 and cathepsin L at pH 6.0 cleavage predictions are shown in a layout of a standard contingency table or confusion matrix format. a) TN= true negative; b) FP = false positive; c) FN= false negative; d) TP= true positive cleavage predictions. For the 'heat diagrams' the predictions are segregated by the P1 anchor residue and are centered (gray) on the overall mean of the four data columns. The associated 'thermometers' are the associated probabilities. Each column represents the results obtained using a different weighting factor. Two-way clusters are formed by the method of Ward [12]. The inset 'shadowgrams' are a histogram-like graphic where a large number (all training and validation cohorts with all weighting factors) of partially transparent distribution kernels are overlayed and thereby simultaneously build up a pattern and density. The vertical axis is probability of that particular classification and the inset is the standard mean, median and quantiles of the underlying distributions.

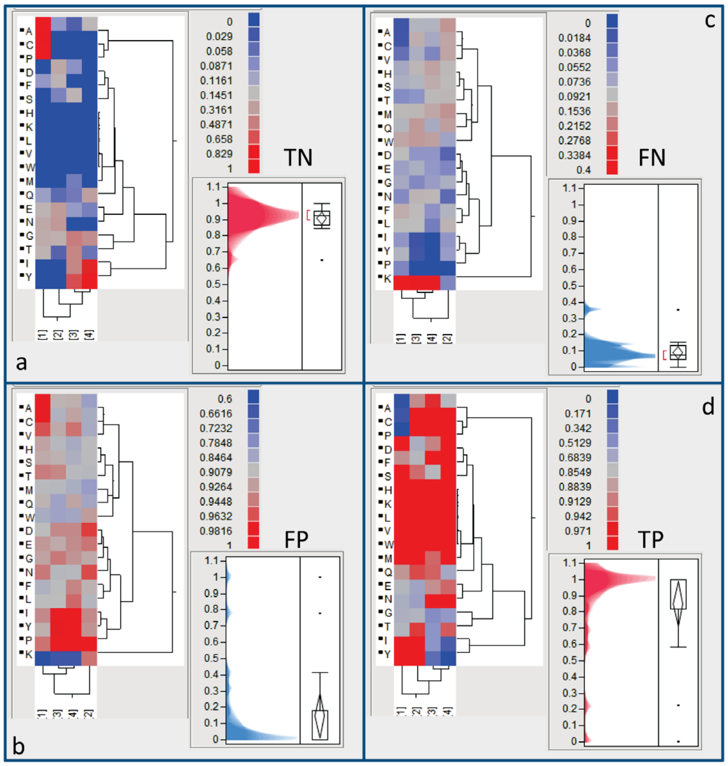

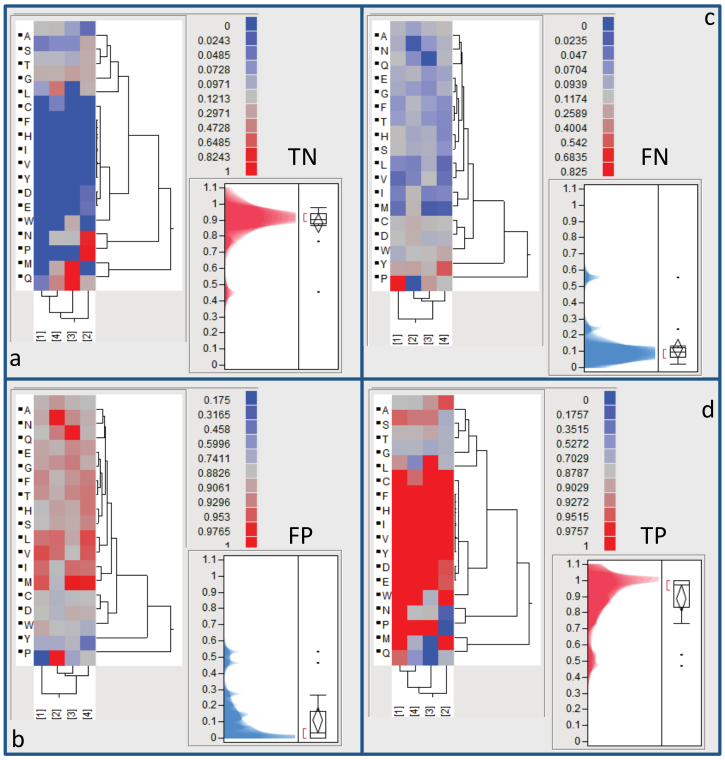

Cathepsin S pH 7.5

Reference List

1. Kleifeld O, Doucet A, auf dem KU, Prudova A, Schilling O, et al. (2010) Isotopic labeling of terminal amines in complex samples identifies protein N-termini and protease cleavage products. NatBiotechnol 28: 281-288.
2. Doucet A, Butler GS, Rodriguez D, Prudova A, Overall CM (2008) Metadegradomics: toward in vivo quantitative degradomics of proteolytic post-translational modifications of the cancer proteome. MolCell Proteomics 7: 1925-1951.
3. auf dem KU, Schilling O (2010) Proteomic techniques and activity-based probes for the system-wide study of proteolysis. Biochimie 92: 1705-1714.
4. Impens F, Colaert N, Helsens K, Plasman K, Van DP, et al. (2010) MS-driven protease substrate degradomics. Proteomics 10: 1284-1296.
5. Agard NJ, Wells JA (2009) Methods for the proteomic identification of protease substrates. CurrOpinChemBiol 13: 503-509.
6. Bremel RD, Homan EJ (2010) An integrated approach to epitope analysis II: A system for proteomic-scale prediction of immunological characteristics. Immunome Res 6: 8.
7. Bremel RD, Homan EJ (2010) An integrated approach to epitope analysis I: Dimensional reduction, visualization and prediction of MHC binding using amino acid principal components and regression approaches. Immunome Res 6: 7.
8. Impens F, Colaert N, Helsens K, Ghesquiere B, Timmerman E, et al. (2010) A quantitative proteomics design for systematic identification of protease cleavage events. MolCell Proteomics 9: 2327-2333.
9. Tholen S, Biniossek ML, Gessler AL, Muller S, Weisser J, et al. (2011) Contribution of cathepsin L to secretome composition and cleavage pattern of mouse embryonic fibroblasts. BiolChem 392: 961-971.
10. Biniossek ML, Nagler DK, Becker-Pauly C, Schilling O (2011) Proteomic identification of protease cleavage sites characterizes prime and non-prime specificity of cysteine cathepsins B, L, and S. JProteomeRes 10: 5363-5373.
11. Bishop CM (1995) Neural Networks for Pattern Recognition. Oxford: Oxford University Press.
12. Ward JH (1963) Hierarchical Grouping to Optimize an Objective Function. JAmStatAssoc 48: 236-244.
